# The Complicate Observations and Multi-Parameter Land Information Constructions on Allied Telemetry Experiment (COMPLICATE)

**DOI:** 10.1371/journal.pone.0137545

**Published:** 2015-09-02

**Authors:** Xin Tian, Zengyuan Li, Erxue Chen, Qinhuo Liu, Guangjian Yan, Jindi Wang, Zheng Niu, Shaojie Zhao, Xin Li, Yong Pang, Zhongbo Su, Christiaan van der Tol, Qingwang Liu, Chaoyang Wu, Qing Xiao, Le Yang, Xihan Mu, Yanchen Bo, Yonghua Qu, Hongmin Zhou, Shuai Gao, Linna Chai, Huaguo Huang, Wenjie Fan, Shihua Li, Junhua Bai, Lingmei Jiang, Ji Zhou

**Affiliations:** 1 Research Institute of Forest Resource Information Techniques, Chinese Academy of Forestry, Beijing, P.R. China; 2 Faculty of Geo-Information Science and Earth Observation, University of Twente, Enschede, The Netherlands; 3 The State Key Laboratory of Remote Sensing Science, Institute of Remote Sensing and Digital Earth, Chinese Academy of Sciences, Beijing, P.R. China; 4 State Key Laboratory of Remote Sensing Science, Research Center for Remote Sensing and GIS, and School of Geography, Beijing Normal University, Beijing, P.R. China; 5 Cold and Arid Regions Environmental and Engineering Research Institute, Chinese Academy of Sciences, Lanzhou, P.R. China; 6 Key Laboratory for Silviculture and Conservation of Ministry of Education, Beijing Forestry University, Beijing, P.R. China; 7 Institute of Remote Sensing and Geographic Information System, Peking University, Beijing, P.R.China; 8 School of Resources and Environment, University of Electronic Science and Technology of China, Chengdu, P.R.China; Beijing University of Posts and Telecommunications, CHINA

## Abstract

The Complicate Observations and Multi-Parameter Land Information Constructions on Allied Telemetry Experiment (COMPLICATE) comprises a network of remote sensing experiments designed to enhance the dynamic analysis and modeling of remotely sensed information for complex land surfaces. Two types of experimental campaigns were established under the framework of COMPLICATE. The first was designed for continuous and elaborate experiments. The experimental strategy helps enhance our understanding of the radiative and scattering mechanisms of soil and vegetation and modeling of remotely sensed information for complex land surfaces. To validate the methodologies and models for dynamic analyses of remote sensing for complex land surfaces, the second campaign consisted of simultaneous satellite-borne, airborne, and ground-based experiments. During field campaigns, several continuous and intensive observations were obtained. Measurements were undertaken to answer key scientific issues, as follows: 1) Determine the characteristics of spatial heterogeneity and the radiative and scattering mechanisms of remote sensing on complex land surfaces. 2) Determine the mechanisms of spatial and temporal scale extensions for remote sensing on complex land surfaces. 3) Determine synergist inversion mechanisms for soil and vegetation parameters using multi-mode remote sensing on complex land surfaces. Here, we introduce the background, the objectives, the experimental designs, the observations and measurements, and the overall advances of COMPLICATE. As a result of the implementation of COMLICATE and for the next several years, we expect to contribute to quantitative remote sensing science and Earth observation techniques.

## Introduction

The term “Complex Land Surface” refers to land-surface systems with complicated landscapes and processes resulting from coexistent landscapes or rugged terrain within an observation footprint. Complex land surfaces are national condition of China and characterized by variations in topography, soil, climate, vegetation and their structural (horizontal and vertical) compositions, including complex terrains, fragmented landscapes, various land types, and diverse land properties [1]. Dynamic analysis and modeling, as well as observations of Earth systems, are the basic tools required for understanding complex land surfaces. As a part of Earth’s observation systems, remote sensing represents ensemble information regarding the radiation characteristics of the pixel or footprint. Dynamic analysis and the modeling of remote sensing information are key for transubstantiating instant observations of remote sensing into space-time continuum information of land surface parameters [2, 3]. Estimations of land surface parameters using remote sensing models have led to numerous achievements. However, due to heterogeneity of mixed pixels, mechanisms of radiative transfer and scattering for complex land surfaces are still not well-known and more investigations are required [4–5]. Recognition of the scale extension of remote sensing information is also a problem [6–7]. Therefore, to achieve better accuracy for the complex land surface parameters, such as the forest vertical distribution of canopy biophysical and biochemical parameters, the hydro-thermal parameters of soil and vegetation, the methodology required for dynamic analysis and modeling of remote sensing information must be further developed. [8–12].

In the context of dynamic analysis and the modeling of remote sensing information, the basic theory of quantitative remote sensing is facing new chances and challenges. Launched by the National Basic Research Program (the 973 Program) of China in 2013, the research program entitled with “Dynamic analysis and modeling of remote sensing information for complex land surface” (hereafter referred to as the 973 Remote Sensing Program for CLS) was designed to meet national strategic demands for improving dynamic analysis and the modeling of remote sensing information using developments in Earth observation techniques. Under the condition of a “complex land surface”, the 973 Remote Sensing Program for CLS focuses on the following: 1) the modeling of the radiative transfer of active and passive (visible, thermal infrared, and microwave) remote sensing; 2) dynamic analysis and modeling of the spatial-temporal scale transfer for multi-scale and longtime-series remote sensing information; 3) the development of a synergistic inversion theory for key land surface parameters using multi-mode remote sensing; 4) establishing a new methodology system for dynamic analysis and the modeling of forest vertical structure information; 5) obtaining three-dimensional information of biophysical and biochemical parameters; and 6) obtaining the hydro-thermal parameters of soil using multi-dimensional remote sensing information.

In order to strengthen the theory and the method for dynamic analysis and the modeling of remote sensing information, and to achieve the scientific objectives of the 973 Remote Sensing Program for CLS, the Complicate Observations and Multi-Parameter Land Information Constructions on Allied Telemetry Experiment (COMPLICATE) was conceived. The COMPLICATE is divided into two campaigns, one campaign that was designed for continuous and elaborate experiments performed at remote sensing test sites and the other designed for simultaneous satellite-borne, airborne, and ground-based experiments conducted on complex land surfaces. Using soil and vegetation as a research entity, COMPLICATE strengthens the established observing infrastructures by performing continuous and elaborate experiments and conducting new and simultaneous space-borne, airborne, and ground-based campaigns over typical complex land surfaces in order to improve dynamic analyses and the modeling of remotely sensed information. Information on the program’s background, its scientific issues and objectives, completed and ongoing campaign measurements, and the current status of COMPLICATE are introduced here.

## Scientific Issues and Objectives

Here, by COMPLICATE, three major scientific issues of quantitative remote sensing are addressed. The first issue is the characterization of spatial heterogeneity and radiative transfer, as well as the scattering mechanisms of remote sensing on complex land surfaces. In the past, the modeling of radiative transfer and scattering processes has been subjected to procedures that describe land surfaces based on ideal hypotheses and realistic scene. Previous experiments have been based on the premise that land surfaces have a well-proportioned distribution [13–14]. As a result, some homogeneous radiative transfer models [15–17] and sparse and discrete geometric-optical models [18–19] have been developed. Although, a series of realistic structural computer simulation models have also emerged [20–21], little is known regarding large-scale directional emissivity [22] and neither radiative transfer nor computer simulator models can precisely depict the dynamic heterogeneity of mixed pixels on complex land surfaces.

As a twin to the optical model, the radiative transfer and scattering model of active microwave remote sensing has a similar developmental history. The model was initialized from the continuous vegetation model [23], then to the layered vegetation model [24], then to the ideal three-dimensional model [25], and then to the real three-dimension model [26]. Trends have recently shifted toward the real three-dimensional coherent backscattering model that is able to determine the horizontal discontinuity and the vertical heterogeneity of terrestrial vegetation. Although a high-resolution version of this type of model has been established [26] using conditions for complex land surfaces, an experiment supporting the development of a middle and low-resolution radar model is indeed necessary. Current vegetation models of passive microwave transfer mechanisms indicate that vegetation is a continuous entity instead of a realistic scene with a three-dimensional heterogeneity. Owing to the complexity of the structure of terrestrial vegetation, passive microwave transfer models of soil-vegetation systems are too complicated to be applied directly. Therefore, parameterization models have been proposed [27–28] that embed both semi-empirical and physical traits. A parameterized soil and vegetation microwave radiative transfer and scattering model that is adaptable to complex land surfaces is urgently required.

Some previously well-established remote sensing experiments initially put their emphases on radiometric calibrations and validations [29–34]. Some authors have stressed modeling radiative transfer and emission mechanisms using active and passive remote sensing for relatively homogeneous land surfaces [35–38]. However, experiments seldom focus on the heterogeneity of remote sensing information within complex land surfaces [39–40]. Innovative COMPLICATE was designed to determine the dynamic characteristics of radiative transfer and the scattering mechanism of heterogeneity for mixed pixels with the goal of advancing radiative and scattering models by conducting elaborate and continuous experiments on complex land surfaces.

The second issue addressed is the mechanism of spatial and temporal scale extensions for remote sensing information on complex land surfaces. Due to multi-scale heterogeneity, land surface complexity inherently incurs uncertainties for pixel information extractions. The spatial scale has been explored for a long period of time [41]. In our previous work, the WATER [39], spatial scale extensions of reflectance, leaf area index (LAI), land surface temperature (LST), vegetation coverage, and additional parameters derived from multi-scale remote sensing data were explored and a general scaling method was developed [42]. However, the applicability of this method has not been tested for complex land surfaces. Without the dynamic integration of a general scaling concept model, the general scaling method must be applied to different land surface parameter estimations using various extensions. Moreover, validations of the various scaling approaches monotonously rely on comparisons between multi-scale remote sensing retrievals. COMPLICATE incorporates campaign measurements with real-scenario computer simulations in order to provide reference data for validations of the general scaling model for complex land surface conditions.

Recently, data assimilation techniques have been applied in order to improve model performance processes using a time-series of remote sensing data [43–45]. However, the overall accuracy, the spatial and temporal continuity, and the spatial and temporal resolutions and consistencies of remote sensing products cannot meet the requirements of scale process models. As indicated by Aires and Prigent [46], innovative techniques combine multi-parameter satellite data, model simulated outputs, and campaign measurements in order to generate refined land surface products. COMPLICATE uses multi-parameter satellite data, dynamic spatial and temporal knowledge, and various continuous experiments to extract dynamic characteristics for the modeling and representations of target terrestrial parameters.

The final issue is determining the synergic inversion mechanisms of soil and vegetation parameters using multi-mode remote sensing on complex land surfaces. Consistently, the accuracy of soil and vegetation parameters is largely impacted by complex terrain [47–49] and by complex vegetation structural heterogeneities [50–51]. At the same time, remote sensing campaigns have evolved into multi-scale, multi-sensor, simultaneous, and synergic data collections, as well as various mechanic model validations [39–40, 52–58]. Additionally, dual-wavelength, multi-spectral, and hyper-spectral Light Detection And Ranging (LiDAR) experiments have also been employed in order to model the vertical distribution of canopy biophysical and biochemical parameters [59–62]. However, most of these results are preliminary [63]. COMPLICATE collects multi-wave LiDAR, interferometric Synthetic Aperture Radar (SAR), stereo optical, multi-angle optical, hyperspectral and infrared data, and passive microwave data in order to determine the synergic inversion mechanisms of soil and vegetation parameters.

The overall objective of COMPLICATE is to pioneer observing techniques to, as follows: 1) obtain dynamic information regarding soil and vegetation of complex land surfaces, 2) develop innovative measuring systems (i.e., new instruments and sensors and alterable full waveform observing platforms, as well as experimental schemes) on multiple scales, and 3) fulfill the multidisciplinary desires of precise dynamic information for remote sensing.

## Experimental Platforms and Sites

**Fig 1:**
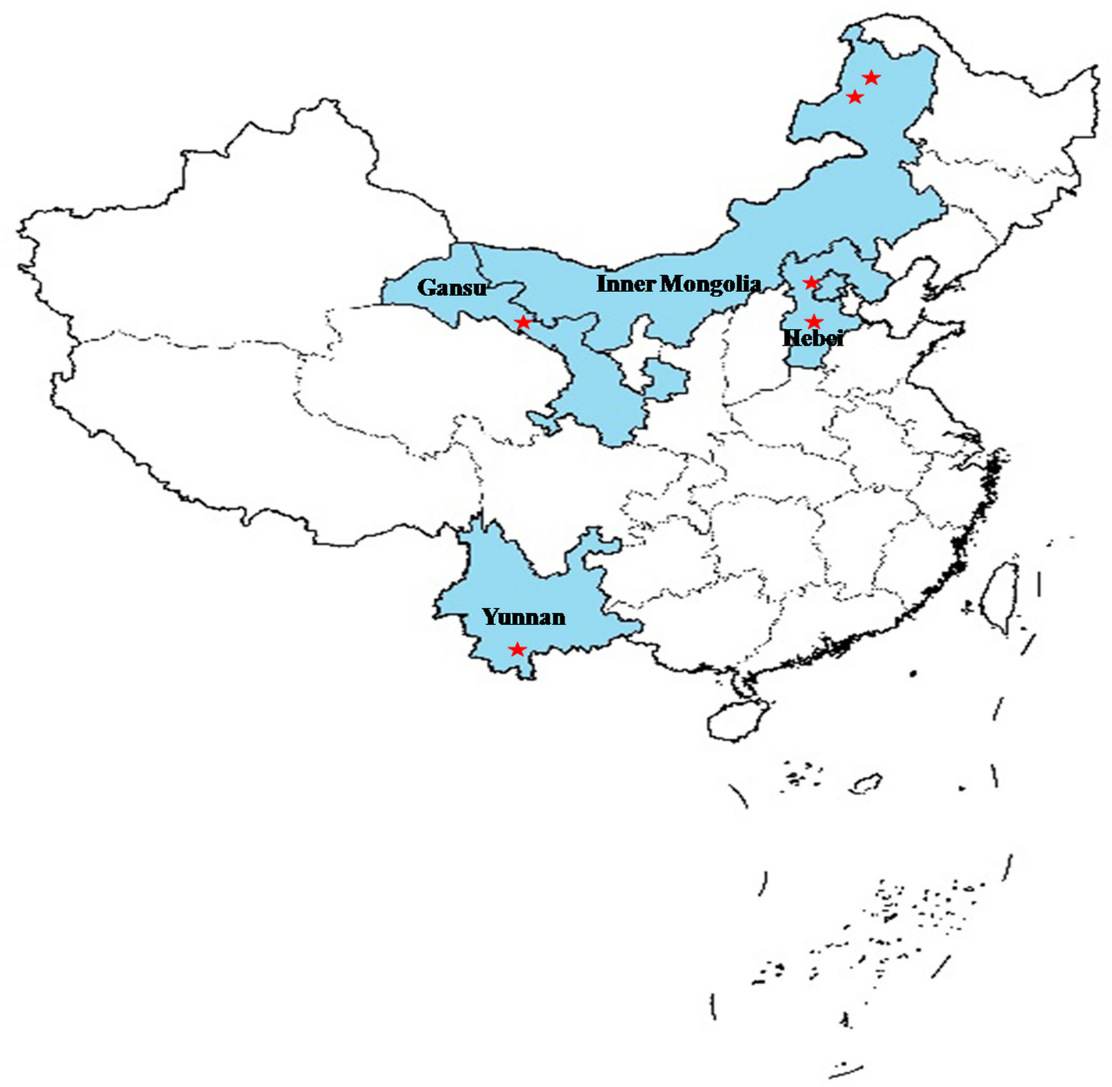
The locations (blue color) of the experimental sites (star symbols) in COMPLICATE.

Since the launch of the 973 Remote Sensing Program for CLS, COMPLICATE has been utilized by two categories of experimental campaigns. The first campaign focuses on the time-series and delicate observations of radiative and scattering components, as well as the parameters of soil and vegetation on complex land surfaces. Such experiments are mainly based on existing and ongoing developments of observation infrastructures established within the remote sensing test sites of Huailai and Baoding in Hebei province, China (Fig 1). The campaign placed an emphasis on the controllability and quantitative interpretations of experimental objectives, as well as measured the accuracy of the instrument using deliberate experimental schemes. Based on the long-term observations of dynamic information for soil and vegetation, the dataset is expected to lead to the development and validation of models.

The other task of COMPLICATE was to conduct comprehensive satellite, airborne, and ground-based observations in order to extract and validate information for the dynamic modeling of soil and vegetation parameters using multi-parameter remote sensing data. During the campaigns, various missions including microwaves within the X and P bands, hyperspectral system, charge-coupled device (CCD), LiDAR and digital camera were located onboard the aircraft or unmanned aerial vehicle (UAV). Multi-source satellite remote sensing data were acquired and several continuous and intensive observations were conducted. Experiments were conducted within several typical complex land surfaces in China, including the Greater Khingan (GK) in Inner Mongolia, the Pu'er (PE) in Yunnan Province, and the Heihe River Basin (HRB) in Gansu Province (Fig 1).

### Ethics Statement

The COMPLICATE did not involve any endangered or protected species. All experiments were conducted at Huailai, Baoding, GK, PE and HRB sites. The Huailai and Baoding sites are managed by the State Key Laboratory of Remote Sensing Science in the Institute of Remote Sensing and Digital Earth of Chinese Academy of Sciences, and the State Key Laboratory of Remote Sensing Science in Research Center for Remote Sensing and GIS and School of Geography of Beijing Normal University, respectively. The permits of the ground measurements in GK, PE and HRB sites were issued by the Forestry Bureau of Greater Khingan of Inner Mengolia, the State Forestry Administration of P.R. China, and the Cold and Arid Regions Environmental and Engineering Research Institute of Chinese Academy of Sciences, respectively. The permits of the airborne flights in GK and PE sites were issued by the local departments of air force of P.R. China. The individual in this manuscript has given written informed consent (as outlined in PLOS consent form) to publish these case details.

### The Huailai Site

**Fig 2:**
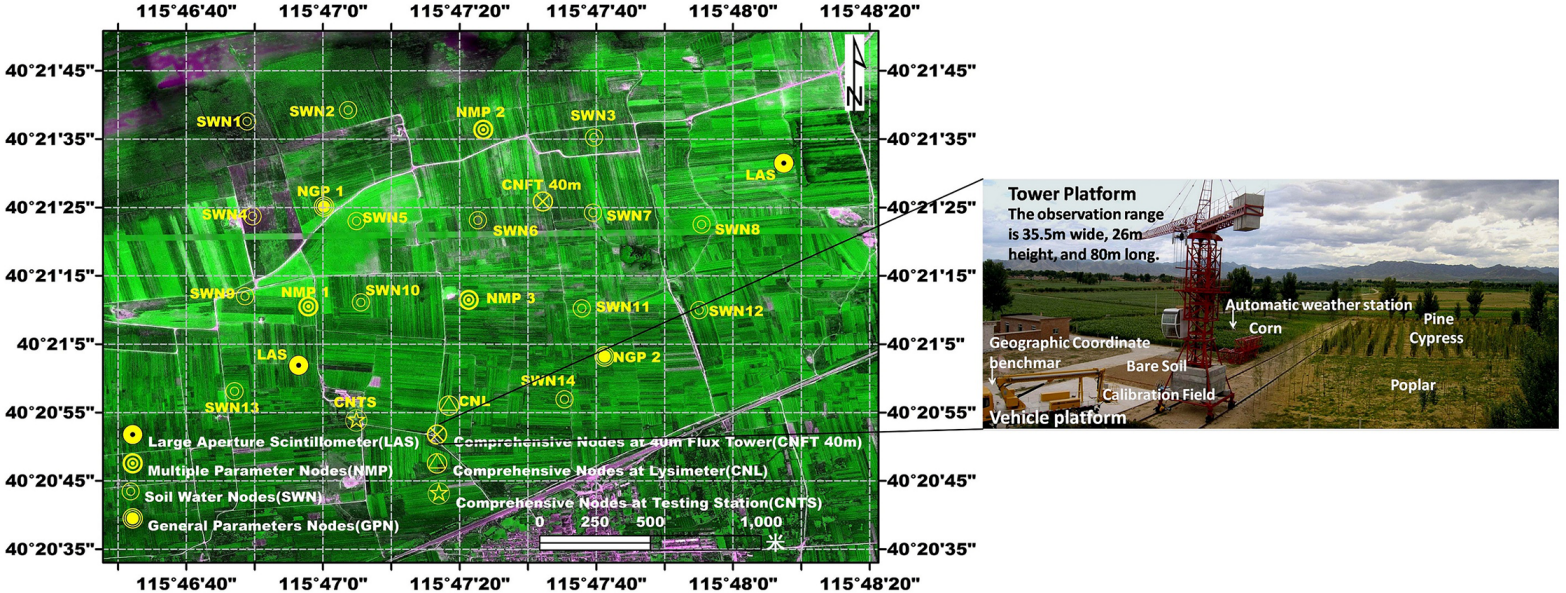
The Huilai experimental test site and the measurement network (the background is a fusion of SPOT-6 and unmanned aerial vehicle image).

At an elevation of 482 m, the Huailai site (40°20′ N, 115°47′ E), established in 2004, is located within the farming-pastoral zone surrounding the border between Hebei province and Beijing city. The surrounding land surfaces were composed of water areas, farmland, highland, and grassland and beach wetland. The site is equipped with advanced instruments such as an aerial lift vehicle and tower, an automatic weather station (AMS), a wireless sensor network, an eddy covariance (EC) system, a large aperture scintillometer, a lysimeter, etc. (Fig 2). The site is an ideal experimental laboratory for the validation of quantitative remote sensing products.

### The Baoding Site

The Baoding site (115°23′ E, 38°42′ N), established in 2009, is located in Qingyuan County of Baoding city within Hebei province. The site has distinct seasons with a windy and dusty spring and autumn, a hot and wet summer, and a cold and snowy winter. As a typical alluvial plain, the surrounding terrain at this site is flat with an altitude below 50 m above sea level.

Observations of the thermal emission of bare soil within microwave and thermal infrared bands were performed in April 2014. A four band ((C-, X-, Ku- and Ka-band) microwave radiometer and a thermal imager were mounted on a hydraulic platform and lifted up to 5.5 m. Apart from above near-surface platforms, automatic ground data loggers were also established for soil and vegetation parameters (i.e., soil and canopy temperature and soil moisture) measurements. Therefore, under the prerequisite of the vertical heterogeneity of vegetation and the artificial complexity of the land surface (wheat, corn, grass, shrub, and forest), the site provides multi-parameter and multi-angle observations of soil and vegetation.

### The Greater Khingan (GK) Site

**Fig 3:**
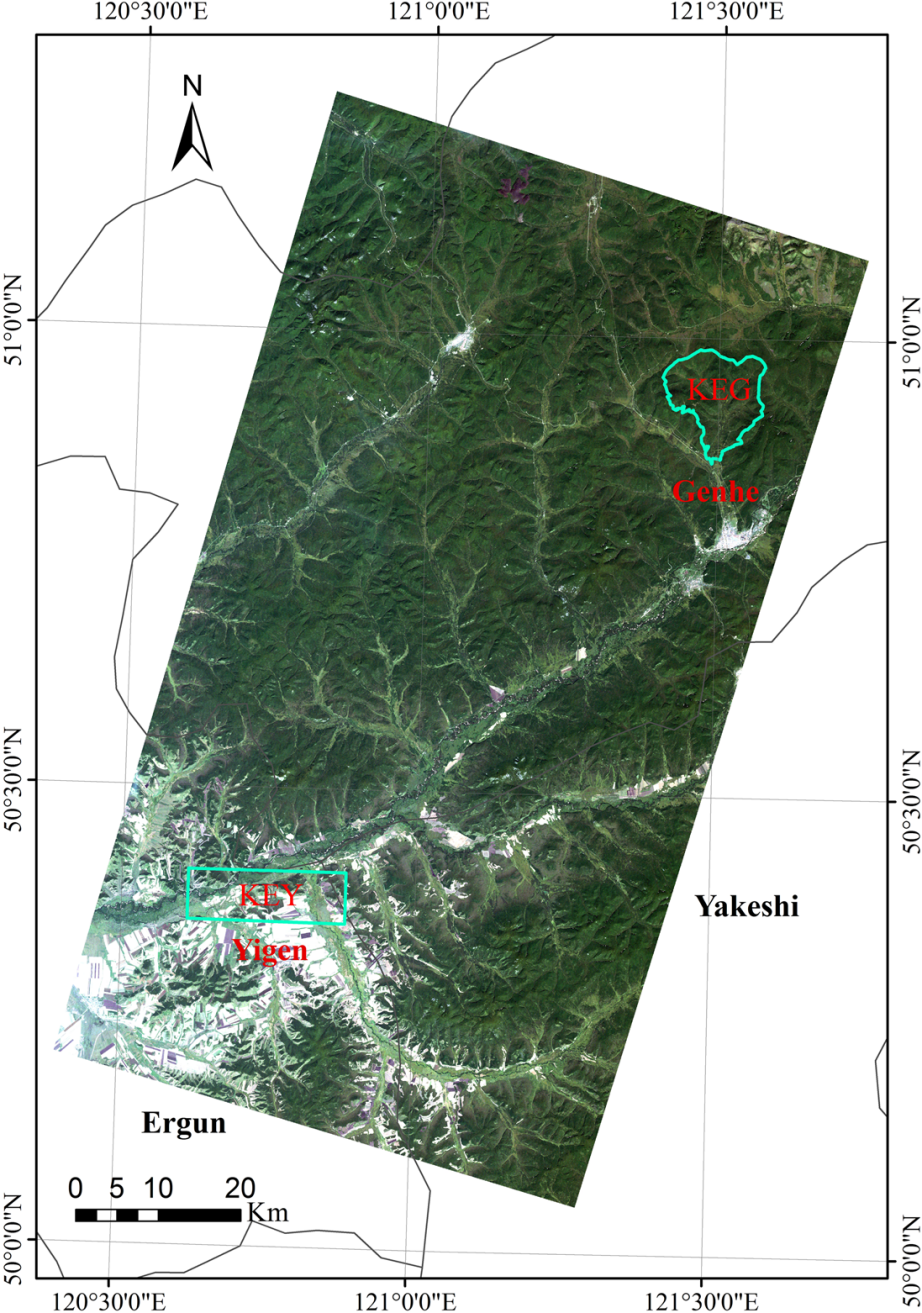
The locations of experimental areas at the Greater Khingan (GK) site (the background is a Landsat Thematic Mapper-5 image).

The GK site is composed of two experimental areas, one located at the Yigen farm (Yigen) (120°36´ to 120°53' E, 50°21' to 50°25' N) and the other at the Genhe forestry reserve (Genhe) (120°12 to 122°55' E, 50°20' to 52°30' N), respectively, both of which belong to the Hulunbeier League in Inner Mongolia. One key area of the Yigen (KEY) and one key area of the Genhe (KEG) have been marked for intensive observations (Fig 3).

Within the high altitude area of Eurasia, Yigen has a cold temperate continental monsoon climate. Mixed within basin plains, numerous gentle slopes are composed of mountain chains with a mean altitude of 900 m. *Betula platyphylla Suk* only exists on shady slopes. With an average altitude of 628 m, Yigen has large farm plots that are cultivated based on recorded crop rotations over several decades. Other land cover types such as grasses, wetlands, water, etc. are mosaicked to the landscapes of Yigen, a complex agroforestry ecosystem.

As the most northern and coldest area in Inner Mongolia, Genhe has a cold and humid temperate forest climate, as well as a continental monsoon climate. With the frost-free period of approximately 70 days, the site is pervaded by permafrost. Located at the western slope of north GK, the site has a hilly topography with slight gradients (80% of them less than 15 degrees) and a mean altitude of approximately 1000 m. The overall geomorphology has a quasi-flat ground and rounded mountains where the tops are flat with similar altitudes. Occupying 75% of the total area, the forest is mainly composed of *Larix gmelinii (Rupr*.*) Rupr*, *Betula platyphylla Suk*, and *Pinus sylvestris var*. *mongolica Litv*. The KEG is the same as for the forest reserve area where one EC station supplemented with an AMS was built in 2008 (Fig 3).

### The Pu'er (PE) Site

**Fig 4:**
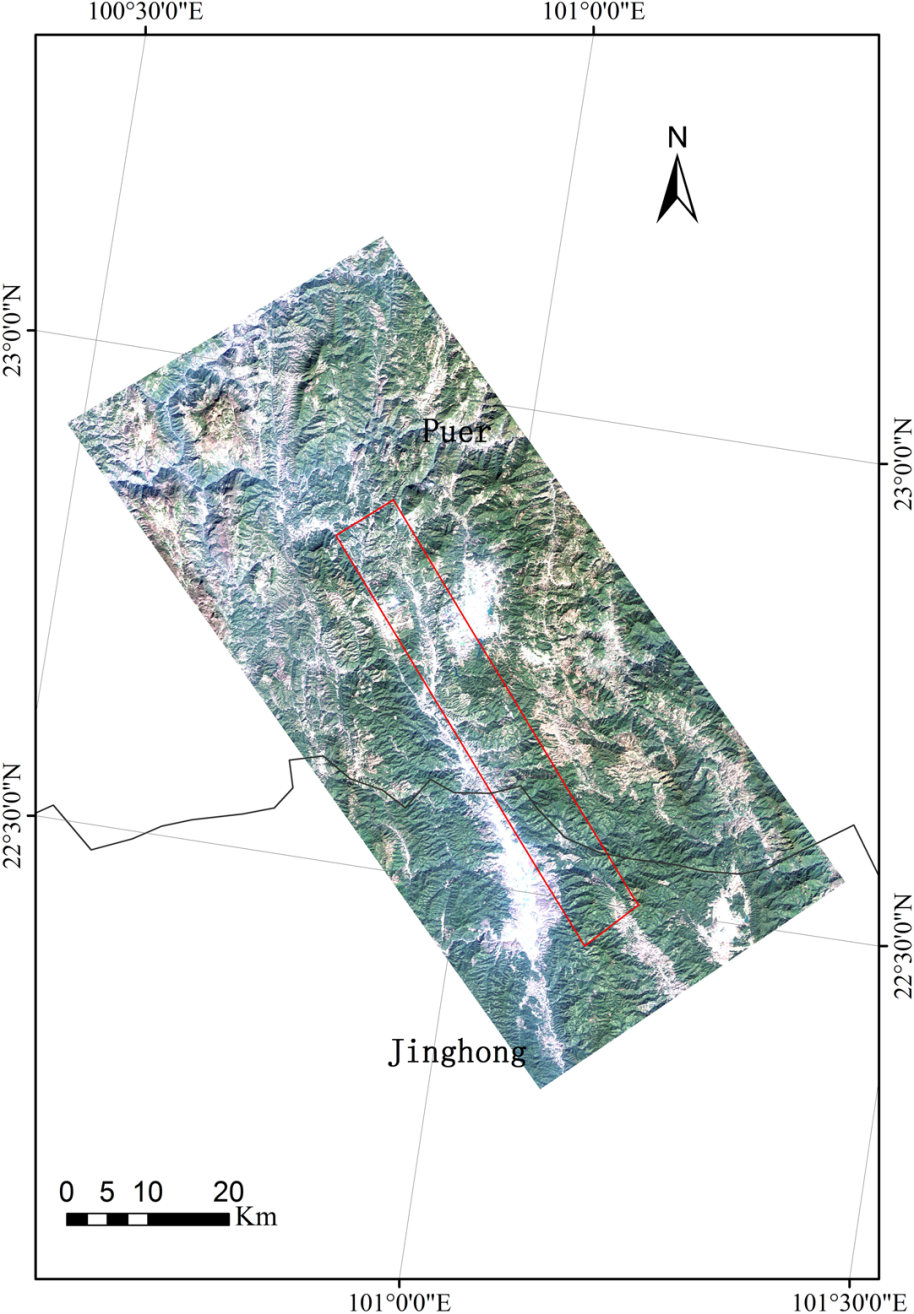
The location of the Pu'er (PE) site (the background is a Landsat Thematic Mapper-5 image).

The PE site is located at the prefecture-level city, Pu'er (99°09' to 102°19' E, 22°02' to 24°50' N). Dominated by mountainous terrain (covering 98.3%), Pu'er has a warm humid subtropical climate. With forest coverage of 62.9% and approximately 15% of total provincial forest stem volume, Pu'er is one of the most important biodiversity source zones in China. With terrain slopes from north to south (3,306 to 376 m), forest vegetation here is vertically distributed and occupies its respective spaces. Forest species display a diversified composition, complicated community structures, and complex land surfaces (Fig 4).

### The Heihe River Basin (HRB) Site

HRB (97°24′ to 102°10′E; 37°41′ to 42°42′ N), located in northwest China, is a well-known cold and arid inland river basin in China. As the second largest inland river basin, it consists of the following three major geomorphic units: the southern Qilian Mountains, the middle Hexi Corridor, and the northern Ejina Basin of the Alxa Highland.

**Fig 5:**
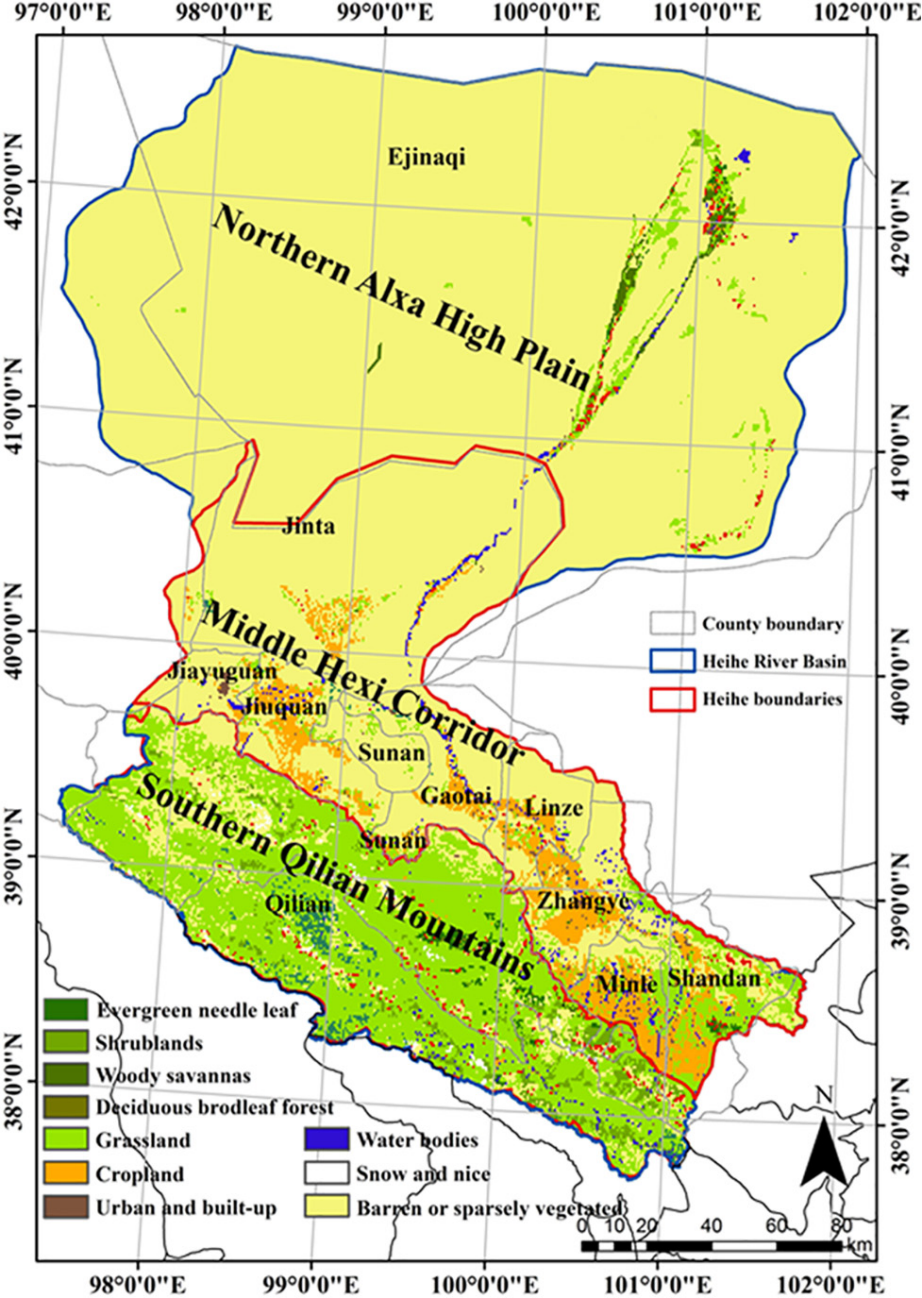
The location and sub-reaches of the Heihe River Basin (HRB) (the background is the landscape map from MODIS data).

The landscapes are various and include a glacier, frozen soil, an alpine meadow, and a forest located within the upper reach (1,500 to 5,500 m); irrigated crops within the middle reach (1,200 to 1,500 m); and a riparian ecosystem and desert (Gobi) within the lower reach (900 to 1,200 m). Influenced by climate and terrain, prevalent vegetation types in the area are mountainous pastures, shrubs, forests, irrigated crops, riparian shrubs, and *Populus euphratica Oliv* forests (Fig 5).

A network of meteorological stations, EC stations, and wireless sensors was built during WATER [39] and HiWATER [40], and a watershed-scale hydrological and ecological observation platform was established. The HRB is an auspicious field experiment site for the development of remotely sensed biophysical parameter models as a result of the complicated variety of environmental factors and the long term implementation of several comprehensive remote sensing campaigns.

## Experiments and Observations

To address the three main scientific issues proposed by the 973 Remote Sensing Program for CLS, COMPLICATE concerns observations for the dynamic analysis and modeling of remotely sensed information on the typical complex land surfaces of China. Complexity is constructed by natural heterogeneity (GK, PE, and HRB) or artificial inhomogeneity (at the Huailai and Baoding sites). COMPLICATE is divided into three types of experiments, including the airborne remote sensing experiment (ARSE), the radiative transfer mechanism experiment (RTME), and the integrated experiment (IE). In particular, the IE consists of the scale extension experiment (SEE) and the synergistic inversion experiment (SIE).

The ARSE enhances the observability of remote sensing on complex land surfaces by acquiring multi-parameter remote sensing data. The experiment complements data support and links ground or near-surface based measurements to satellite remote sensing observations. The RTME stresses radiative and scattering mechanisms and the dynamic modeling of remote sensing information. The SEE is concentrated on the spatial and temporal scale extension of remote sensing information. The SIE focuses on forest vertical structure information (physical, biophysical, and biochemical) and hydro-thermal parameters for soil and vegetation. In other words, the RTME provides the basic dataset as well as derivative radiative and scattering transfer models for the scale extension methods developed from the SEE, both that improve the applicability of synergistic inversion models calibrated and validated by the SIE.

### The Airborne Remote Sensing Experiment (ARSE)

#### The Airborne SAR Experiment

Airborne SAR missions were flown over the GK site with coverage of more than 5,619 km^2^ during September of 2013. The airborne SAR system, namely CASMSAR, was integrated using a SAR data acquisition system, a SAR mapping workstation, and a SAR data preprocessing and distribution system. The system is the first airborne SAR mapping system in China and was developed by the Chinese Academy of Surveying and Mapping (CASM) in collaboration with several research institutes in China [64].

The airborne SAR data acquisition system is comprised of a flight control and navigation subsystem, a dual-antenna X-band interferometer, and a fully polarimetric P-band SAR (Table 1). SAR images with a resolution of 0.5–5 m at an altitude from 3 to 10 km can be acquired. As a result, scales of 1:10,000, 1:25,000, and 1:50,000 within images and products can be produced. The integrated SAR missions with an interferometric X-band and a full-polarimetirc P-band SAR flown over the GK area were the first flights in China.

**Table 1:** The major parameters of CASMSAR.

Parameter	X-SAR Sensor	P-SAR Sensor
Operating frequency (GHz)	9.6	600
Available bandwidth (MHz)	400	200
PRF range (Hz)	1000–4000	500–800
Pulse peak power (kW)	4.0	1.0
Pulse width range (us)	8–22	15–70
Interferometric baseline (m)	2.198	N/A
Polarization mode	HH	HH, HV, VH, VV
Ground resolution (m)	0.5/1.0/2.5/5.0	1.0/2.5/5.0
Swath width (km)	~2-~12	~3-~11.5
Incidence angle (°)	~37-~63	~33-~53
Onboard electronics	2 cabinets, 2 antennas	1 cabinet, 1 antenna

**Fig 6:**
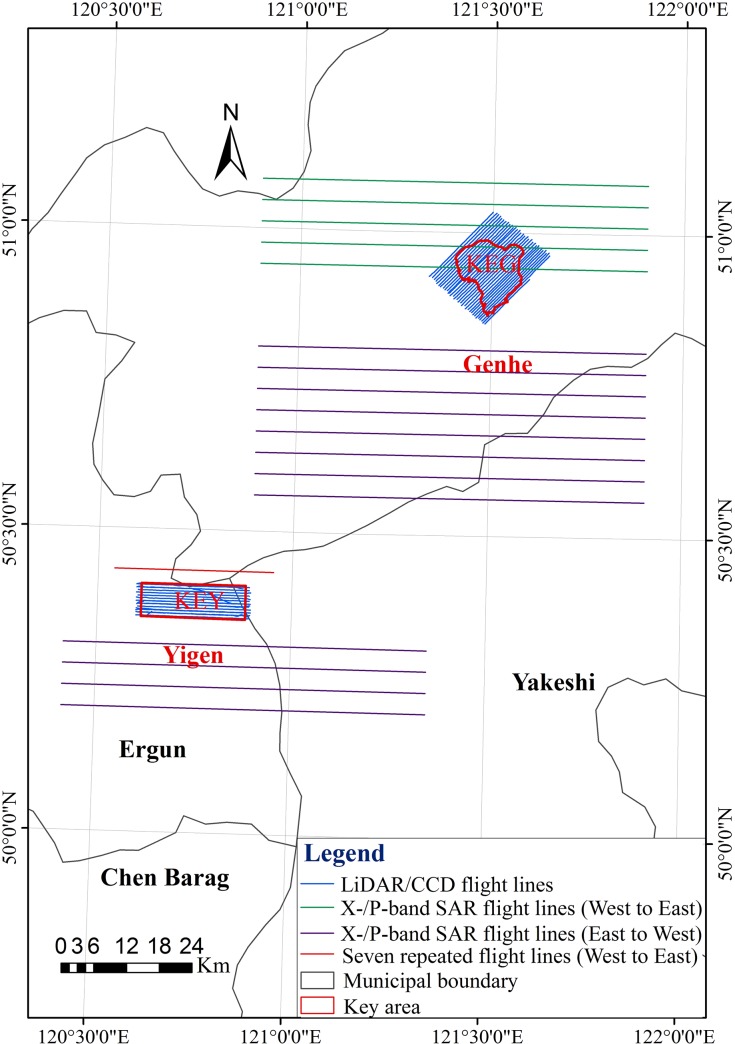
The overall flight areas and lines of the CASMSAR and LiDAR+CCD missions at the KG site.

**Fig 7:**
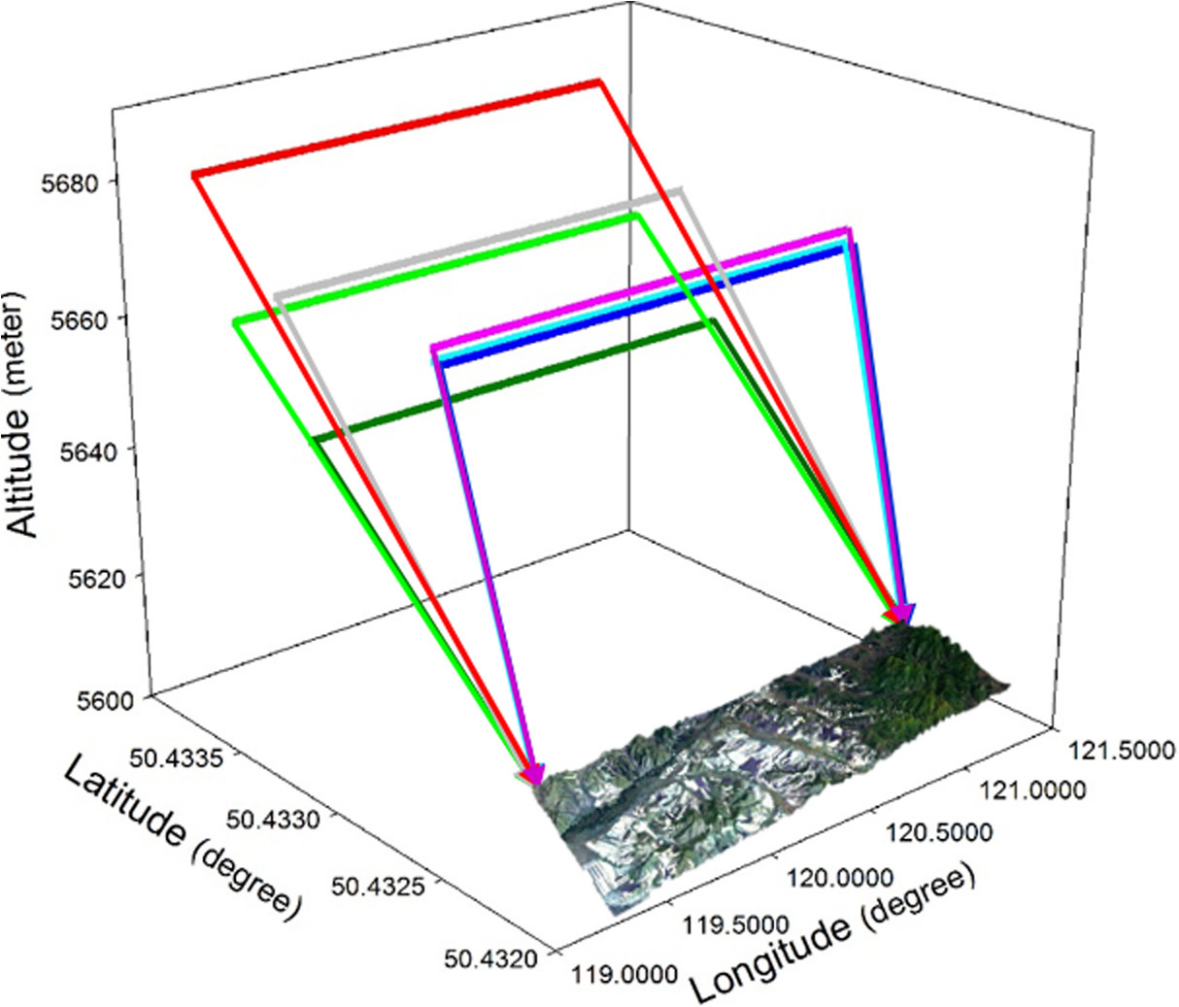
The seven repeated flight lines of CASMSAR (the background is a Landsat Thematic Mapper-5 image).

The flight region included the Yigen area, the KEY, and the Genhe areas (Fig 6). In total, 24 flight lines (12 lines from east to west and 12 lines from west to east) were flown (Fig 7). Seven repeated flights (from west to east) flown over the key area of Yigen were implemented in order to obtain polarimetric coherence tomography information within the forest. With a flying height of 5,670 to 5,810 m, 0.5 to 1 m resolution interferometric X-band data and 1 m resolution polarimetirc P-band data were acquired. Within this region, a high resolution (5 m) DEM was produced. Additionally, the very high resolution (0.5 to 1.0 m) interferometric, polarimetric, and backscattering characteristics of dual band SAR data was helpful for developing reliable models for alleviating the terrain relief, simulating the radiative transfer and the large-scale scene, deriving forest structural information, and estimating soil and vegetation parameters.

#### The Airborne LiDAR Experiment

Two airborne LiDAR experiments have been conducted since the summer of 2012. The first, with an onboard LiDAR (Leica ALS60, Leica Geosystem AG, Heerbrugg, Switzerland) and CCD-Leica RCD105, Leica Geosystem AG, Heerbrugg, Switzerland) (Table 2) was conducted over the GK site, covering KEY (~130 km^2^) and KEG (~230 km^2^), from August to September of 2012. Following 153 flight lines and employed on the Yun-5 aircraft, the LiDAR+CCD mission acquired cloud point data with an average density of 8.0 points/m^2^ and a 0.2 m resolution for CCD data. LiDAR and CCD missions provided information for modeling forest and crop structural and biogeophysical parameters (forest density, vegetation height, canopy width and shape, canopy coverage, LAI, LAD, clumping index, etc.), and terrain factors (altitude, slope, aspect, roughness, etc.). With a very high resolution (0.5 m) and with accurate observations within key experimental areas, in the context of complex land surfaces, LiDAR+CCD missions will help advance remote sensing models for the following: 1) radiative transfer and scattering simulations; 2) scale transformations; 3) synergistic inversions of forest structural, biophysical, and biochemical information; and 4) soil and vegetation parameters.

**Table 2:** The major parameters for the LiDAR+CCD missions.

**LiDAR: Leica ALS60**			
Wavelength	1064 nm	Laser beam divergence	0.3 mrad
Laser pulse length	3.5 ns	Scan angle range	±30°
Maximum laser pulse	200 KHz	Maximum scanning speed	200 lines/s
Repetition rate			
Waveform sampling	1 ns	Vertical accuracy	0.15 m
**CCD: LeicaRCD105**			
Pixel resolution		8964×6716(60 million pixels)	
Imaging sensor size		43.30mm×53.78mm	
Pixel size		6.8 um	
Radiometric resolution		16 bits	
Imaging focal length		50 mm	

**Fig 8:**
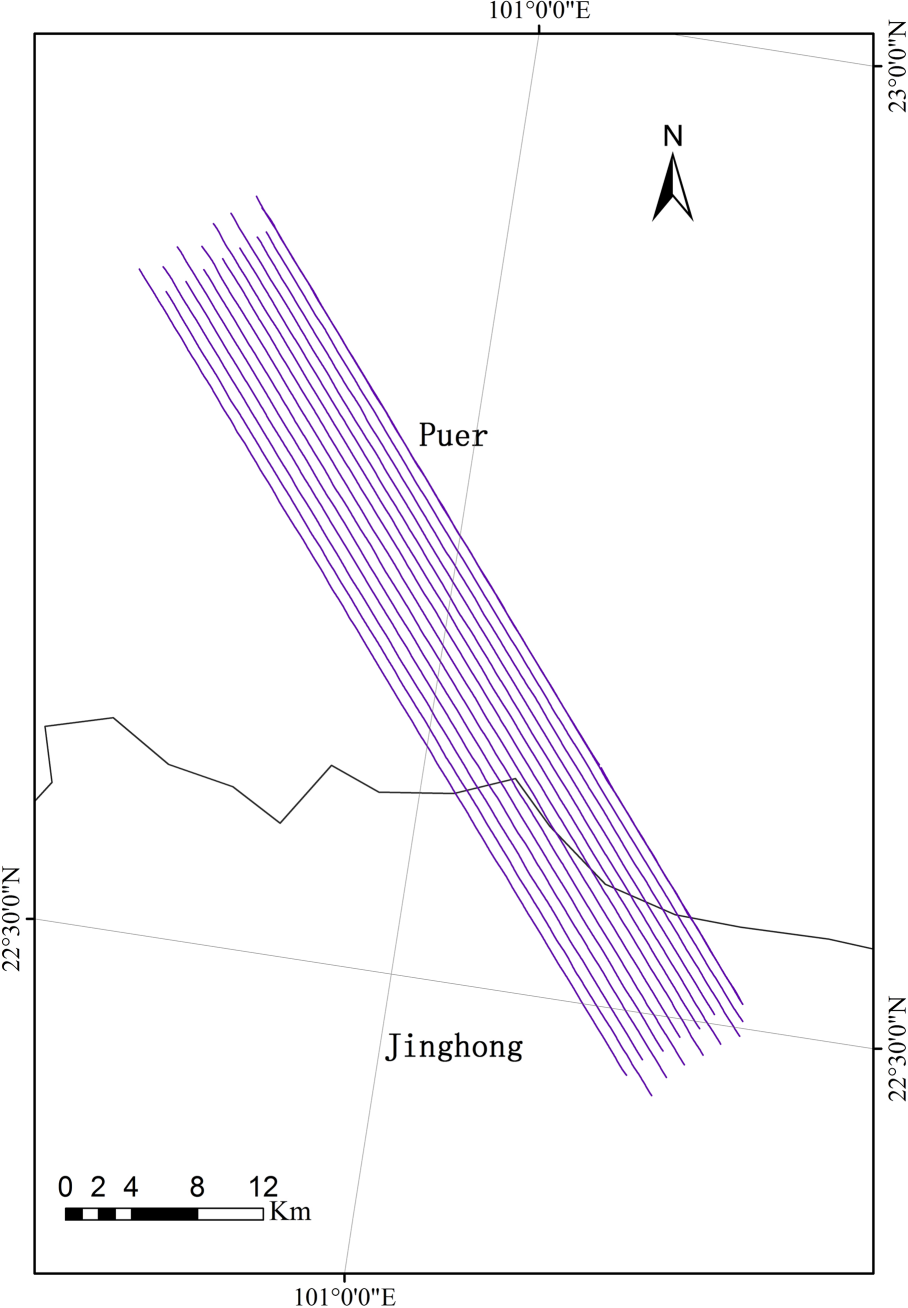
The flight areas of the LiCHy missions.

Additional integrated LiDAR+CCD+Hyperspectral (LiCHy) missions (Table 3) were composed of a Riegl LMS-Q680i (Riegl Laser Measurement Systems GmbH., Horn, Austria), a DigiCAM-60 (IGI mbH., Kreuztal, Germany), and an AISA Eagle II (Spectral Imaging Ltd., Oulu, Finland) that acquired airborne multi-parameter data (including cloud point data with an average density of 2.0 points/m^2^, hyperspectral data with a 1.1 m spatial resolution and a 9.2 nm spectral resolution from 398 to 994 nm, and CCD data with a 0.2 m resolution) over the PE site. Missions were flown over the site during April 2014 onboard the Yun-12 aircraft, by 14 flight lines with coverage of 510 km^2^ (Fig 8). Using the merits of advanced missions, the LiCHy system is able to observe specific details of topography as well as soil and vegetation, especially for the vertical distribution of canopy biophysical and biochemical information. Therefore, COMPLICATE rises to the scientific challenges and the development of relevant remote sensing models for complex land surfaces with various vegetation types and species, as well as a diverse topography.

**Table 3:** The major parameters of the LiCHy system.

**LiDAR: Riegl LMS-Q680i**			
Wavelength	1550 nm	Laser beam divergence	0.3 mrad
Laser pulse length	3 ns	Scan angle range	±30°
Maximum laser pulse	400 KHz	Maximum scanning speed	200 lines/s
Repetition rate			
Waveform sampling	1 ns	Vertical accuracy	0.15 m
**CCD: DigiCAM-60**			
Pixel resolution		8964×6716(60 million pixels)	
Imaging sensor size		43.30mm×53.78mm	
Pixel size		6 um	
Radiometric resolution		16 bits	
Imaging focal length		50 mm	
**Hyperspectral:AISA Eagle II**			
Spectral range	400–970 nm	Spatial pixels	1024
Focal length	18.1mm	Spectral resolution	3.3nm
Field of View	37.7°	Maximum bands	488

### The Radiative Transfer Mechanism Experiment (RTME)

The RTME aims to develop, calibrate, and validate new radiative transfer models in both the optical and microwave regions for mixed land covers such as crop-forests or corn-wheat. Therefore, the RTME focuses on observations of the radiation distribution (including the energy balance), the bidirectional reflectance distribution function (BRDF), the albedo, the reflectance, the brightness temperature, radiation fluxes (latent and sensible heat), and other related parameters (meteorological parameters, soil moisture, LAI, vegetation height, biomass and coverage, canopy height, fraction of absorbed photosynthetically active radiation (FAPAR), etc.). Based on the ongoing upgrade of infrastructures located at the Huailai site, the system integrates current ground-based remote sensing facilities and techniques in order to develop innovative methods for synergizing ground-based remote sensing observations and measurements of relevant parameters.

**Fig 9:**
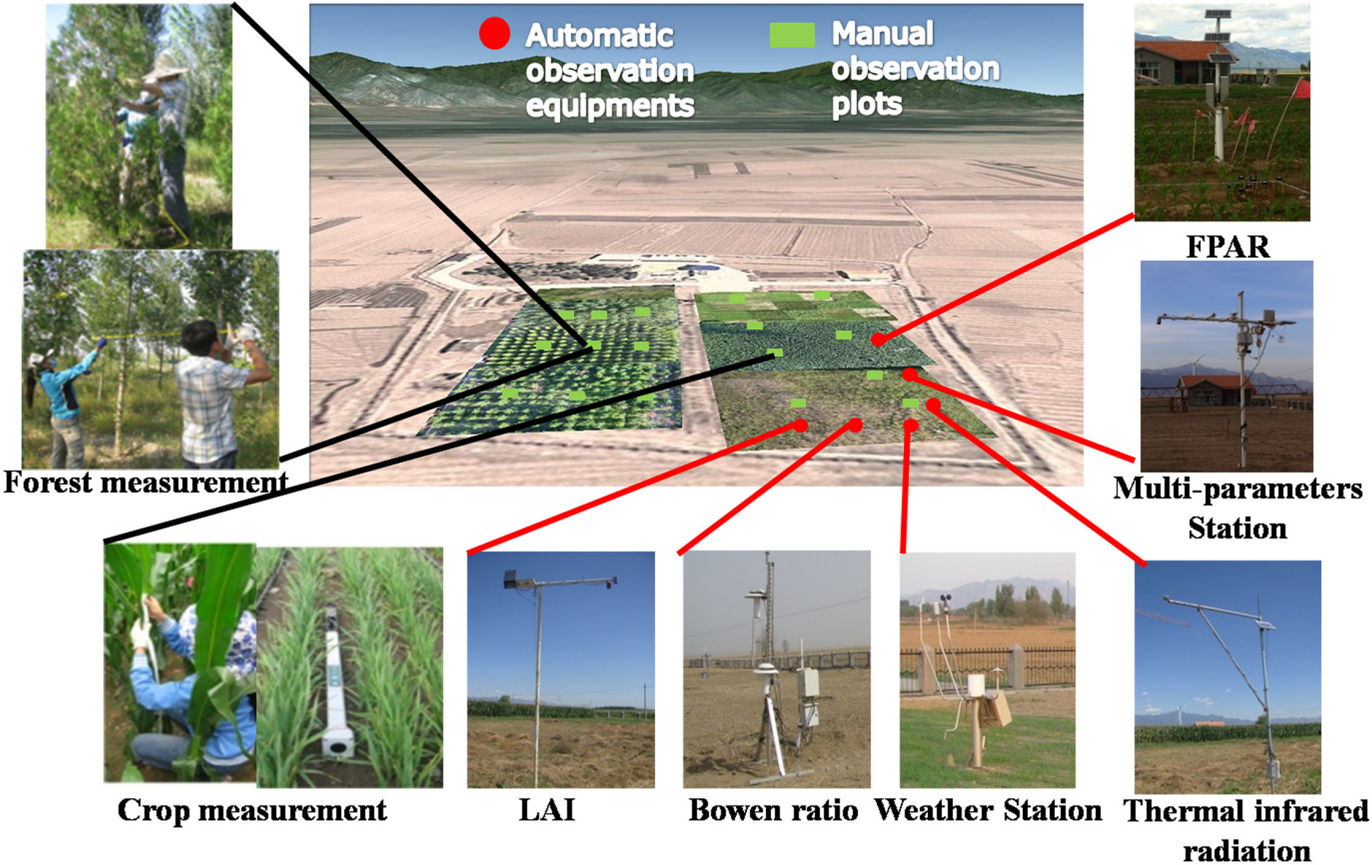
The test range of symmetrical radiation characteristics.

With goals of the construction and optimization of radiative transfer models such as heterogeneous models for the BRDF, thermal, and SAR over complex land surfaces with two or more end members and based on research of the sampling theory and method, the experiment established a test range of symmetrical radiation characteristics (Fig 9) and compliant measurements were conducted (Table 4). The near-surface remotely sensed observation platform and the multiband sensors were integrated into the field-based remote sensing subsystem. The subsystem was designed to be adjustable in order to acquire various end members as well as multi-angle and multi-temporal observations, and plays an essential role in connecting ground measurements to satellite remote sensing observations.

**Table 4:** The major instruments within the test range.

Instrument	Land Cover Type	Frequency	Observation Items
Camera	Crops	Two times/days	LAI
Bowen ratio	Wheat	Ten minutes/once	ET
Meteorologicalstation	Wheat	One hour/once	Multi-layer wind speed & direction, temperature, humidity, soil temperature
CNR4	Corn	<6s(63%); <18s(95%)	Downwelling shortwave radiation, upward longwave radiation
Ultraviolet radiation meter	Corn	1s	Downwelling and upward radiation of UV
Infrared radiation thermometer	Corn	1.4s	Brightness temperature
FAPAR system	Corn	5s	FAPAR

### Integrated Experiment (IE)

The IE consisted of the SEE, and the SIE. The IE aims to improve the applicability of multi-source remote sensing within integrated scaling extensions and the synergistic inversions of forest vertical structure information and hydro-thermal parameters for soil and vegetation under conditions of complex land surfaces.

Since 2013, in association with ARSE and RTME experiments, IE has been performed at all sites of the 973 Remote Sensing Program for CLS. Major parameters jointly measured by the IE groups are listed in Table 5.

**Table 5:** The major parameters jointly measured by the IE groups.

Scale	Observation Items	Key Instruments
Site	Atmosphere: multilayer air temperature, wind speed/direction, humidity, heat flux, pressure, four component radiation, PAR, LST, precipitation, snow depth;Soil: Multilayer moisture/temperature, roughness roughness, reflectance; EC.	AMS, radio soundings, time domain reflectometry, EC system,
Vegetation	Size, density, defoliation ratio, inclination angle distribution, tree core, water content, clumping index,	ASD, total station, integrating sphere, canopy spectroradiometer
component	spectral reflectance and transmittance, orientation, chlorophyll and C/N ratio,	SPAD,
Individual	Species, position, DBH, height, first	Total station, holometer,
plant	live branch height, crown shape and size	ground-based LiDAR
Vegetation	Position, dominant tree species, tree density, LAI, LAD, albedo, FAPAR, productivity, brightness temperature, forest canopy	GPS, LAI 2000, LAI2002, Hemiview, TRAC, LAINet, LAINet, quantum meter,
plot	coverage fraction, underlying vegetation coverage fraction,woody-to-total area ratio, multi-component spectrum,	albedometer, TMMR, UAV camera, imaging spectrometer, canopy spectroradiometer and ground-based LiDAR,
Large scale	Land cover types, terrain information quasi-real virtual 3-D scene of forest	GPS, airborne CCD/ LiDAR/ SAR and computer simulation

#### The Scale Extension Experiment (SEE)

The SEE was composed of spatial and temporal scale extension experiments (SSEE and TSEE, respectively). The SSEE was mainly performed over KEG during 2013. We designed multi-scale comprehensive observations for vegetation structural parameters in order to calibrate and validate the spatial scale transformation models. Specifically, the SSEE provided calibration and validation data for the development of general conceptual scaling models, the development of parameterized physical scaling models, and an analysis of the optimal scale for analyzing scaling effects and scaling calibrations for indirect ground measurements on real vegetation structural parameters, as well as for the development and validation of scale transformation models for retrieving vegetation structural parameters.

**Fig 10:**
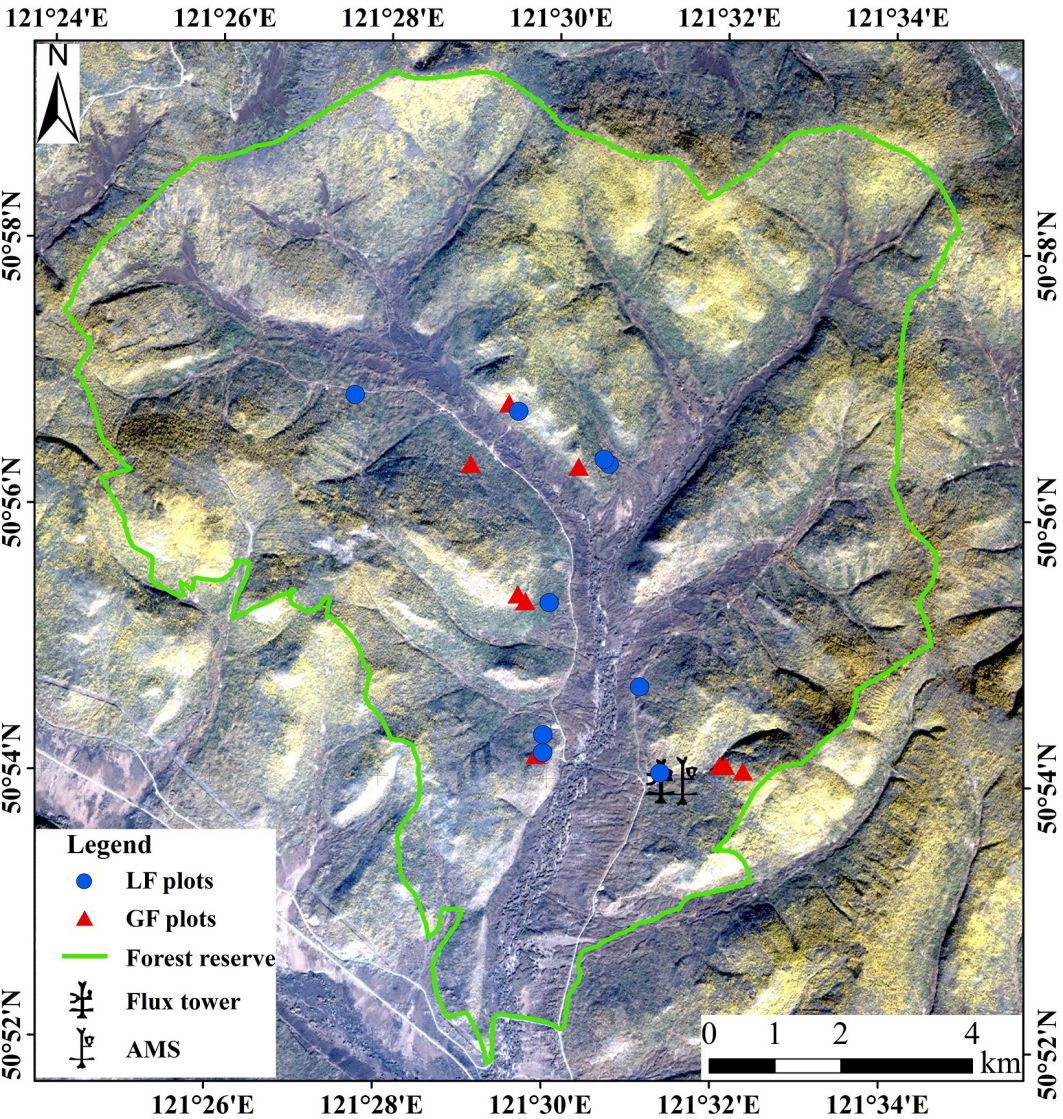
The locations of LF and GF plots within the KEG (the background is a SPOT-6 image).

In collaboration with additional field experiments (RTME, TSEE, and SIE) and various sampling schemes (different observational elements and sampling schemes), the SSEE collected measurements that were comparable or proportional to the pixel size of multi-scale remote sensing data. In the context of complex land surfaces distributed over comparative slopes (the shady and sunny slopes) of the KEG, two types of forest plots were designed. One was designed for ground-based LiDAR scanning and intensive forest inventory (hereafter referred to as LF plots) and the other was designed for general forest inventory in regions with highly complex terrains (hereafter referred to as GF plots) (Fig 10). The plots contained particular land surface conditions (with various forest types, stand ages, stand densities, canopy closures, aspects, gradients, etc.) of the same size, 45 × 45 m^2^ and was equally divided into nine subplots.

Vegetation parameters, including LAI, FAPAR, FVC, clumping index, woody-to-total area ratio, spectral reflectance, and transmittance were intensively observed at leaf, crown, plot, and large scales. Such structural and biogeophysical parameters of vegetation are critical for improving scaling models. The spatial scale gap between ground measurements and satellite remote sensing was expected to be bridged by use of very high resolution airborne data (SAR, LiDAR+CCD) and the digital camera image from unmanned missions.

The temporal scale was also a key issue of the scaling transformation. Designed for exploring temporal scale transformation methods, the TSEE is largely based on time-continuous observations consisting of an automatic data collection network. The network provides time-series observing data that supports the model dynamic characteristics of vegetation parameters and improves methods for extracting the temporal characteristics of typical vegetation parameters and the dynamic knowledge base.

Jointly conducted with additional experiments, the TSEE was performed over the Huailai site, KEY, and KEG during 2013. We measured vegetation structural parameters (such as LAI, height, etc.), and surface albedo and reflectance using automatic data collection systems. LAI and forest canopy coverage were observed using LAI2000, TRACK, HemiView, and manual measurements in order to improve LAI retrieval using comparative validations.

At the Huailai site, the albedo instrument has been located on the 40 m tower since July of 2013. Since that time it has collected land surface albedo data within the large supersite (2×2 km^2^) in order to provide support to the LAI data logger network (LAINet) observations. Relying on weather conditions and the arrangement of the observational period, a canopy spectroradiometer (SVC-1024) was used to measure the corn canopy at the LAINet sensor nodes in each plot (30×30 m^2^) and in one intensive observation plot (30×30 m^2^) surrounding the tower. For LAINet measurements, fifteen plots each with three to four sensor nodes were distributed within the supersite, and in total, 65 nodes were installed. Instruments were applied to LAI measurements from 29 June to 14 September.

At the Yigen site, in a similar manner, a canopy spectrum (SVC-1024) and LAI measurements (LAI2000) were performed at ten large sites (each with 1×1 km^2^) at Yigen. At each site, the LAI of each vegetation type was measured twice then the overall LAI was calculated by weighting the measurements of each vegetation type according to their area fractions and total area. At the Genhe site, one LAINet with 15 nodes was installed at one LF. The measurement was performed from 14 August to 15 October.

Based on these measurements, the following scientific questions were explored: 1) Are we able to obtain expressions for multi-scale mapping time-series land surface parameters using ground measurements? 2) Is it possible to capture the clumping effects of various scales on land surface vegetation parameters using a wireless instrument net?

#### The Synergistic Inversion Experiment (SIE)

In addition to scaling studies, synergistic inversions also require the vegetation, biophysical, and biochemical parameters listed in Table 5.

The following experiments were conducted in 2013:

(1) The experiments of forest vertical structure

These experiments were essentially concerned with parameters at the following three scales: 1) the tree component, 2) the individual tree, and 3) the forest plot. Five forest inventories were conducted: two at KEY, two at KEG, and one at PE. Five sizes were measured for the forests: 10×10 m^2^ (43 plots at the PE site and 80 plots at the KEY site), 30×30 m^2^ (59 plots at the KEG site), 45×45 m^2^ (18 plots at the KEG, with each containing nine 15×15 m^2^ sub-plots), and forest cycloidal sample with a diameter of 10 to 15 m (39 plots at the PE site). Specifically, at the KEY site, 80 plots comprised a forest sampling stripe with a 10 m width and an 800 m length, whereas the forests were relatively homogeneous with one tree species, *Betula platyphylla Suk*.

At the tree component scale, we measured size, density, defoliation ratio, inclination angle distribution, the chlorophyll and water content of the leaf, and leaf clumping index, as well as spectral reflectance and transmittance of the leaf, bark, and crown for the selected trees with different conditions (age, species). At the individual tree scale, the tree species, the position, the height, the first live branch height, the diameter at breast height (DBH), and the tree crown shape and size were intensively investigated for each tree except for those with a DBH less than 5 cm. At the plot scale, the positions of the four corners, the dominant tree species, tree density, LAI, albedo, FAPAR, forest canopy coverage fraction, and underlying vegetation coverage fraction were intensively performed.

For comparative analysis and calibration, LAI was measured using various instruments including LAI 2000, LAI2200, TRACK, HemiView, fish-eye camera, and LAINet. Individual tree structural and biogeophysical parameters, the precise positions, and the 3-D structural information of the tree and its components (branch, trunk), as well as the reflectance and transmittance of the leaf, bark, and crown were measured using the total station, the ground-based LiDAR scanning instrument, the analytical spectral devise (ASD), and the optics integrating sphere, respectively. The total station and the ground-based LiDAR scanning system were also determined within some plots and placed at multiple sites in order to obtain a panoramic perspective of each tree. With the assistance of airborne remote sensing data and computer simulations, and the large scale quasi-real, virtual 3-D scenes of the forest can be established. Therefore, using multi-scale 3-D forest information and terrain information (e.g., the DSM and the DEM), algorithms for radiometric and scattering compensation on terrain relief for multi-source remote sensing data will hopefully be improved.

To support forest dynamic AGB modeling, a forest productivity investigation was also conducted within 28 plots located at the KEG site. Three trees were selected for each species at each diameter (five classes at each plot of 5 to 10, 10 to 15, 15 to 20, 20 to 25, and>25 cm) for measurements within (or near if there was no sufficient sample) each plot. The tree core was extracted at two orthogonal directions at a height of 1.30 meters where the DBH was measured using a vegetative cone in order to estimate the annual net primary productivity (ANPP) of the plot. ANPP is a good indicator for validating forest AGB dynamic modeling behavior for long term simulations.

(2) The experiments for the vertical distribution of canopy biophysical and biochemical parameters

The biophysical and biochemical experiments consist of multi-angle observations by active and passive optical instruments (ground-based LiDAR and the near-surface hyperspectral imager) on multilayer canopy biophysical and biochemical parameters. Based on field measurements, backward waveforms and spectral characteristics (from LiDAR and the imaging spectrometer, respectively) of the vegetation’s biophysical (leaf inclination, branch orientation, canopy height, LAD, LAI) and biochemical information (chlorophyll, water content, C/N ratio), the retrieval algorithms for the vertical distributions of corresponding parameters are being developed at three scale levels (leaf, tree, and plot). Afterward, the experiment is expected to provide support for the basic theory, database, and prototype required for innovations of multi-wave LiDAR instruments with the merits of hyperspectral and laser detection on vegetation vertical characteristics.

Under the prerequisite of complex land surfaces, these experiments were performed over various forest areas and croplands. At the KEG site, a Terrestrial Laser Scanner (TLS) and hyperspectral imagery instruments were used in order to observe the vertical distribution of biophysical and biochemical parameters over two LF plots (LF5 and LF6). The Leica HDS C10 ground 3D laser scanner was adopted in order to acquire a point cloud with a high scanning rate of 0.05 m at a distance of 100 m. Spectral images were acquired from various horizontal and vertical scanning angles using the SOC710 imaging spectrometer, the ultimate portable hyperspectral imager with 128 bands from 400 to 1,000 nm. Ground measurements of tree height, LAI, and biochemical parameters (chlorophyll, water content, lignin) were also performed.

To observe vertical information of the canopy and underlying vegetation using the Leica HDS C10 and the SOC710 at the KEY site covered by mature wheat and barley, two plots with sizes of 10×10 m^2^ were selected. Besides these two plots, an additional 15 plots (10×10 m^2^) of wheat and barley were measured for biophysical and biochemical parameters including the vegetation height, the canopy FAPAR, chlorophyll, and the water content of the leaf.

At the PE site, a forest biophysical and biochemical investigation was conducted. Three forests and five cash crop (tea tree, leechee, and coffee tree) plots with sizes of 10×10 m^2^ were scanned by Leica HDS C10 and SOC710. Five forest plots with sizes of 10×10 m^2^ were scanned with the ground-based LiDAR instrument and 39 plots of the same size were measured for LAI (using LAI2000, LAI2200 and fish-eye camera), chlorophyll (using Soil and Plant Analyzer Development, SPAD), and water content (using a dry weighting instrument).

(3) The experiments of the hydro-thermal parameters of soil and vegetation

These experiments were emphasized using in-situ, hydro-thermal measurements of soil and vegetation through the implementation of elaborative and controlled measurements. Here, the hydro-thermal parameters refer to soil temperature and moisture (including the surface soil freeze/thaw status), the LST, the vegetation canopy temperature, and the vegetation water content. The microwave radiation characteristics of typical land surfaces (i.e., bare soil, winter wheat, corn, forest, snow, etc.) were measured using Truck-mounted Multi-frequency Microwave Radiometers (TMMR) at Baoding site. The TMMR is characterized by multi-frequency (C-, X-, Ku- and Ka-bands with a center frequency of 6.925, 10.65, 18.7 and 36.5 GHz, respectively), dual polarization (V/H), and a multi-angle view. For the duration of the experiment, the structural and physical parameters of the target objectives (LAI, leaf inclination, stem orientation, vegetation height, density, dielectric constant, correlation length, and emissivity) have been frequently obtained from field measurements. Integrating these measurements with active and passive remote sensing observations, synergetic inversion models for the hydro-thermal parameters of soil and vegetation are expected to be improved.

**Fig 11:**
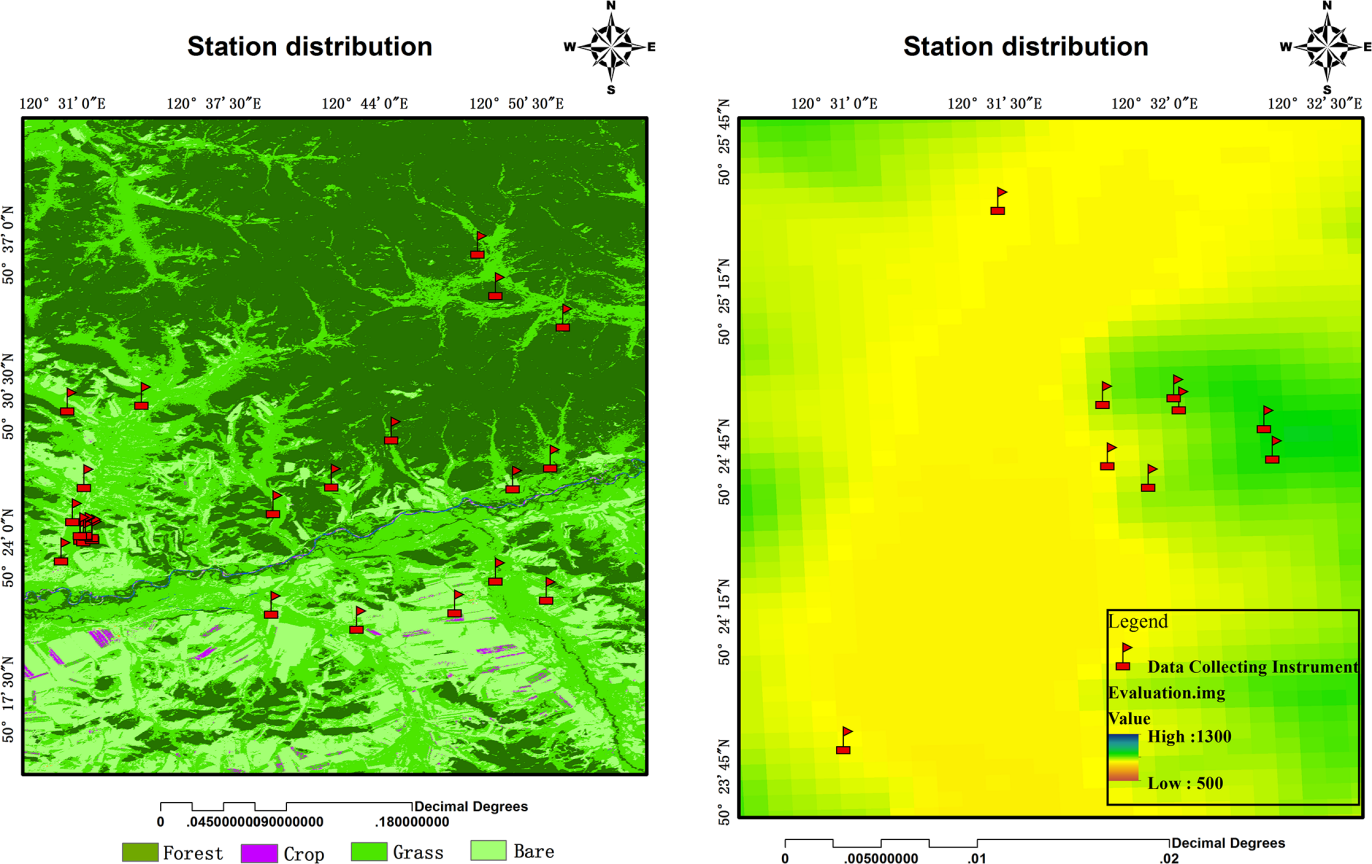
The locations of the SoilNet nodes at the GK site (the left panel provides the land cover types and the right panel provides the elevation of the partial enlargement).

To determine the sensitivity of the soil moisture and temperature inversion algorithm, a soil data logger network (SoilNET) was setup at the GK site (Fig 11) from 11 July to 8 October, 2013 in order to measure soil moisture and temperature. SoilNET consists of 26 sensor nodes distributed over various land cover types (forests, croplands, grasslands, shrubs, and bare soil) with diverse elevation heights. For each node, three temperature probes were inserted into the soil at three depths (3 cm, 5 cm, and 10 cm) and one moisture probe was inserted into the soil at a depth of 5 cm.

**Fig 12:**
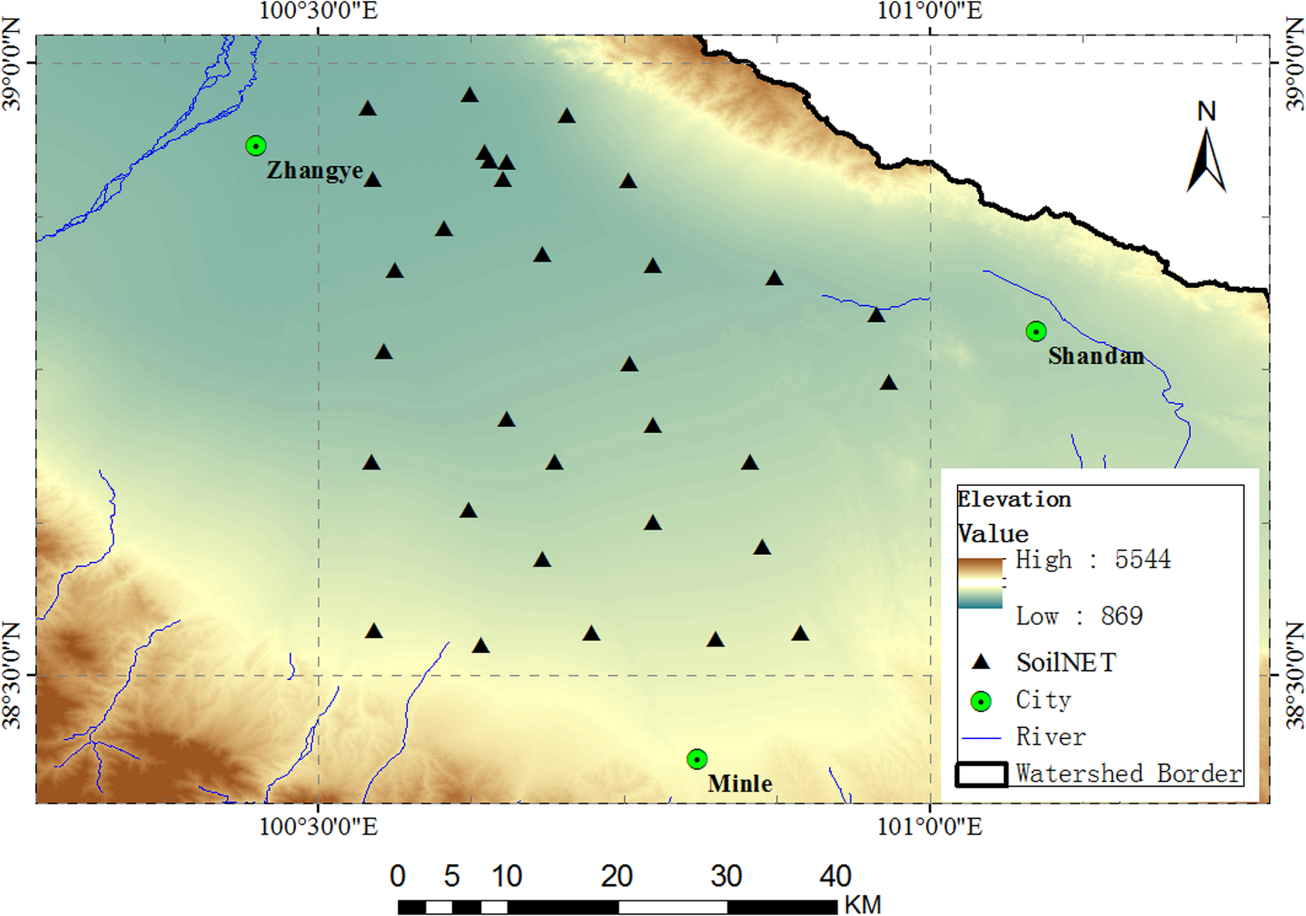
The distribution of SoilNET nodes over the upper reaches of the HRB.

Another SoilNET with 32 nodes was installed over the upper reaches of the HRB (Fig 12), where land covers were mainly arid with little or no vegetation and composed of farmland. The elevation of this area ranged from 1,400 to 2,500 meters. The spatial scale of this area was comparable to the sizes of the SMOS, AMSR-E, and AMSR2 pixels. Measurements began on 5 October and ended on 1 March of 2014.

Two additional SoilNET nodes were also placed within a gravel desert (GB) site (41.949°N, 100.897°E) and a partially vegetated area within the Sidaoqiao site (41.990°N, 101.134°E) in the Ejin Banner Oasis, the lower reaches of the HRB. The GB site is largely homogenous. The landscapes of the Sidaoqiao site are composed of barren land, cropland, *Populus euphratica*, and *Tamarix chinensis*. At this site, two key areas of 100 × 100 m^2^ were set and all of the trees within key areas were scanned from the horizontal and vertical directions using a Leica ScanStation C10 laser scanner. Component temperatures were observed using a Testo thermal camera and two Fluke thermal infrared radiometers. Worth mentioning is that this ground measurement was jointly conducted within the HiWATER [40]. Atmospheric profiles that provided detailed vertical distributions of temperature, humidity, and pressure were measured using radio soundings when Terra ASTER passed over.

To analyze and validate the feasibility of the winter wheat optical depth inversion algorithm which aids in the retrieval of the water content of vegetation, TMMR was used from 18 May to 20 June 2013 to measure the brightness temperature of winter wheat continuously at the Baoding site during the grain-filling stage. Following calibration, absolute errors of TMMR measurements were less than 2 K.

**Fig 13:**
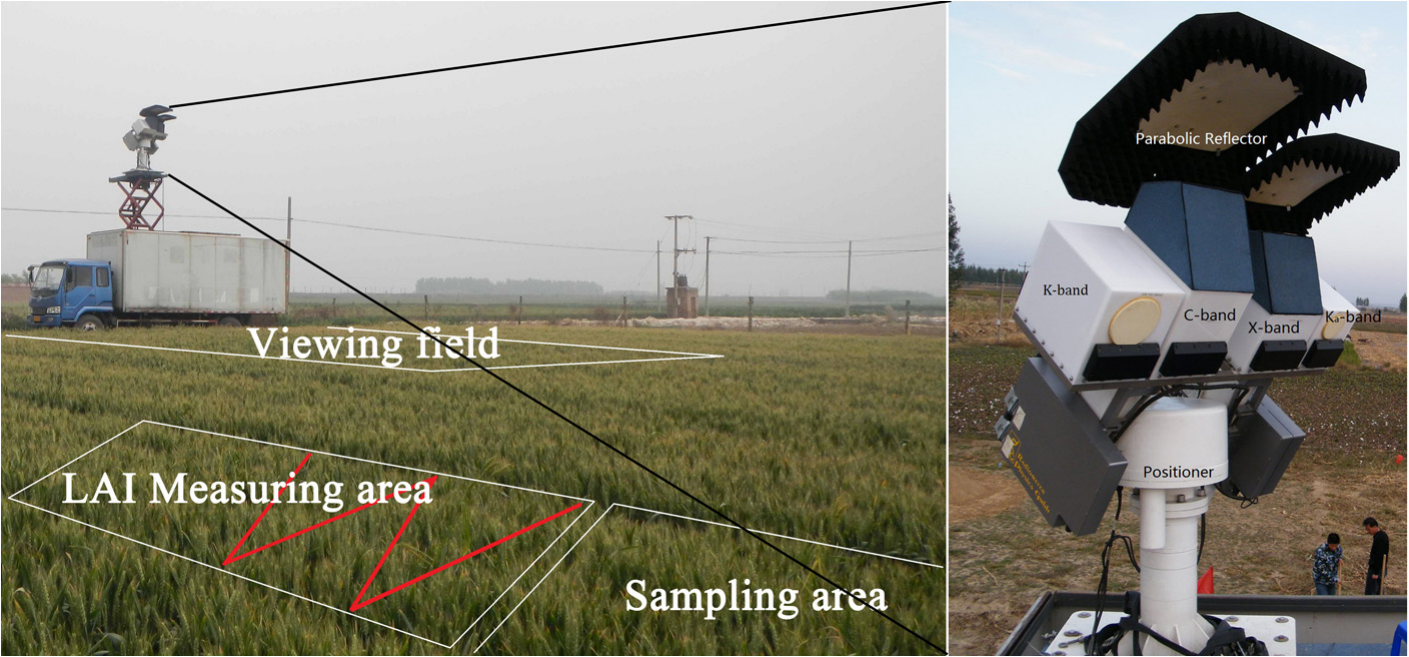
The observation scene (left) of the TMMR instrument (right).

With a footprint size of 3 × 4 m^2^, TMMR was performed for observations at different azimuths in order to determine the impacts of the row structure of wheat fields (with a size of 30 × 40 m^2^) on brightness temperature measurements. For the duration, soil temperature, soil moisture, structural parameters (height, length, and width) and the water content of the wheat stem, leaf, and ear, the LAI, the reflectance of soil, the wheat canopy and its composition, were measured each day. LAI was measured along a "Z"-shaped route (Fig 13).

## Satellite Data Acquisition

To fulfill the scientific objectives of the 973 Remote Sensing Program for CLS, the collection of satellite data was also an important part of COMPLICATE. Multi-sensor data was collected, including multi-spectral, hyperspectral optical data, LiDAR data, and active and passive microwave data (Table 6). Data were obtained without charge under the framework of the data sharing program and international cooperation, or by commercial purchase. Commercial data were almost always obtained during periods of airborne campaigns and corresponding ground measurements with the exception of SPOT-6, which was obtained at the conclusion of the experiment due to extremely cloudy conditions.

**Table 6:** The major satellite remote sensing data acquired in COMPLICATE.

Type	Remote Sensors	Observation Aims	Time
Very high resolution multi-spectral (1~5 m)	IKONOS, ALOS PRISM, Worldview-2, QuickBird, SPOT-5/6 HRG, KH-4B ZY03 and GF-1	Mapping experimental area forest structure parameters	Archived and experimental period (AEP)
High resolution multi-spectral (10~100 m)	ASTER, ALOS AVNIR-2, TM TM-8, ZY-1 02C, HJ-1A/B	Reflectance, albedo, LST, biogeophysical parameters	AEP
Medium-resolution multi-spectral (>100 m)	MERIS, and MODIS	Reflectance, albedo, LST, biogeophysical parameters	AEP
Multi-angle	MISR	BRDF and biogeophysical parameters	Archived
Thermal	AATSR	LST	Archived
SAR	Envisat ASAR, ALOS PALSAR Radarsat-2, TerraSAR-X,	Forest structure parameters, soil moisture, soil freeze/thaw status	AEP
Passive microwave	SMOS, AMSR-E and AMSR2 and FY-3/MWRI	Soil moisture, soil freeze/thaw status	AEP
LiDAR	ICESAT/GLAS	Forest structure parameters	Archived

## Summary

COMPLICATE has a five year (2013 to 2017) design of experimental progressions that accompanies implementations of the 973 Remote Sensing Program for CLS. The program seeks to improve the observability, understanding, and applicability of remotely sensed dynamic modeling information for complex land surfaces. Since the launch of the program, COMPLICATE has strengthened established observing infrastructures in order to perform continuous and elaborate experiments and has also conducted new and simultaneous airborne, space-borne, and ground-based campaigns over complex land surfaces.

By performing comprehensive, multiscale, continuous and elaborate experiments, COMPLICATE has collected a multiscale but spatiotemporally consistent data set for the dynamic analysis and modeling of remotely sensed information for retrieving soil-vegetation parameters under the condition of complex land surface. The 973 Remote Sensing Program for CLS addresses scientific issues that have hindered the applicability of quantitative remote sensing modeling for a long period of time. The overall background, scientific issues and objectives, the status of completed and ongoing campaign measurements, and the current status of COMPLICATE have been addressed here.

As compared with previous remote sensing campaigns, COMPLICATE seeks to understand complex land surfaces using complete information integration. Based on the data set from COMPLICATE, dynamic analyses and calibrations and validations of models have been conducted.

The remaining experimental plan is currently undergoing discussions and improvements, and we have much confidences that a fruitful outcome for quantitative remote sensing studies, focusing on dynamic analyses and the modeling of remote sensing for complex land surfaces, will be achieved and led by implementing COMPLICATE.
